# Applications to augment patient care for Internal Medicine specialists: a position paper from the EFIM working group on telemedicine, innovative technologies & digital health

**DOI:** 10.3389/fpubh.2024.1370555

**Published:** 2024-06-28

**Authors:** F. Pietrantonio, M. Florczak, S. Kuhn, K. Kärberg, T. Leung, I. Said Criado, S. Sikorski, M. Ruggeri, A. Signorini, F. Rosiello, C. Drago, A. Vinci, V. Barreto, N. Montano, D. Dicker, R. Gomez Huelgas

**Affiliations:** ^1^Medical Area Department, Internal Medicine Unit, Castelli Hospital, Rome, Italy; ^2^Saint Camillus International University of Health Sciences, Rome, Italy; ^3^Department of Immunology, Transplantology and Internal Medicine. Medical University of Warsaw, Warsaw, Poland; ^4^Institute of Digital Medicine, University Hospital of Giessen and Marburg, Phillips-University Marburg, Marburg, Germany; ^5^Department of Internal Medicine, Institute of Clinical Medicine, University of Tartu, Tartu, Estonia; ^6^Care and Public Health Research Institute, Maastricht University, Maastricht, Netherlands; ^7^Department of Internal Medicine (Adjunct), Southern Illinois University School of Medicine, Springfield, IL, United States; ^8^Palliative Care Unit, Internal Medicine Department, Pontevedra-El Salnés Healthcare Area, Institute of Healthcare Research, Vigo, Spain; ^9^Institute of Law Studies, Faculty of Law and Administration, Cardinal Stefan Wyszyński University in Warsaw, Warsaw, Poland; ^10^Department of Public Health and Infectious Disease, Sapienza-University of Rome, Rome, Italy; ^11^University Niccolò Cusano. Department of Economics, Psichology and Communication Sciences, Rome, Italy; ^12^Local Health Authority ASL Roma 1, Health Management Unit, Rome, Italy; ^13^Pedro Hispano Hospital, Porto, Portugal; ^14^Department of Clinical Sciences and Community Health, University of Milan, Milan, Italy; ^15^Internal Medicine Department and Obesity Clinic, Hasharon Hospital, Rabin Medical Center, Sackler School of Medicine, Tel-Aviv University, Tel Aviv-Yafo, Israel; ^16^Internal Medicine Department, Hospital Regional Universitario de Málaga, Instituto de Investigación Biomédica de Málaga (IBIMA), University of Málaga, Málaga, Spain

**Keywords:** e-health, digital medicine, Internal Medicine, tele health, digital health

## Abstract

Telemedicine applications present virtually limitless prospects for innovating and enhancing established and new models of patient care in the field of Internal Medicine. Although there is a wide range of innovative technological solutions in Europe, there are overarching elements associated with such technologies when applied to the practices of Internal Medicine specialists. The European Federation of Internal Medicine (EFIM) strongly advocates for active leadership and influence from the Internal Medicine societies and specialist physicians across Europe in the development and application of telemedicine and digital technologies in healthcare. This position paper’s conclusions were drawn via Delphi method, which was developed collaboratively from July 2021 to December 2023. The panel, consisting of experts in clinical medicine, public health, health economics and statistics, assessed various aspects related to telemedicine. Participants assigned scores on a Likert scale reflecting perceived value and potential risks. The findings were consolidated in a comprehensive checklist aligning with relevant literature and a SWOT analysis. Specifically, key issues that need to be addressed include promoting the professional development of e-health competencies in the healthcare and medical workforce, using educational campaigns to promote digital literacy among patients and caregivers, designing and implementing telemedicine applications tailored to local conditions and needs and considering the ethical and legal contexts under which these applications are employed. Importantly, there is currently no consensus on care models or standardized protocols among European Internal Medicine specialists regarding the utilization of telemedicine. This position paper aims to outline the opportunities and challenges associated with the application of telemedicine in Internal Medical practice in Europe.

## 1 Main objective

In various medical settings in Europe, numerous innovative technological solutions have been applied. This creates a specific need to comprehensively evaluate the applications of these solutions in the field of Internal Medicine. The European Federation of Internal Medicine (EFIM) deeply encourages Internal Medicine societies and internists in Europe to actively engage in Innovative Technologies by means of a written statement. The aim of this position paper is to provide Internal Medicine specialists as well as health professionals, managers, and decision makers, with a framework that highlights the best practices implemented in different European countries. This document serves as a resource, delineating issues and terminology and suggesting recommendations. However, it is not intended to override regulatory or credentialing recommendations and guidelines. Instead, it aims to align with and support the professional and ethical standards of the profession. The paper suggests potential future developments of patients’ and clinicians’ behavior and their interactions, illustrating four possible scenarios.

## 2 Introduction

Telemedicine, as defined by the World Health Organization (WHO), entails the delivery of healthcare services by healthcare professionals employing Information and Communication Technology (ICT) where distance is a critical factor (1, 2). Telemedicine facilitates the exchange of pertinent information related to diagnosis, treatment, prevention, research, and disease assessment (1, 2). In addition, significant advancements in information technology, the advent of high-speed internet, and the proliferation of smartphones over the past decade have greatly enhanced the accessibility of telemedicine services. The terms “telemedicine” and “telehealth” are often used interchangeably, although they can have distinct meanings. Telemedicine refers to “the provision of healthcare services, including remote care and online pharmacies, through the use of information and communication technologies, in situations where the health professional and the patient (or several health professionals) are not in the same location” (3), while telehealth includes a wide range of health promotion and education toward a healthy lifestyle, which also includes providing remote care (4), such as telemedicine, telenursing, teletherapy, and telepsychology. The goals are similar for each: to improve access to healthcare services.

Telemedicine has a positive impact on patient health behavior, medication adherence, and quality of life due to its efficiency and cost-effectiveness. Moreover, the utilization of telemedicine in the field of Internal Medicine can improve the management of various chronic conditions and clinical outcomes. The adoption and implementation of evidence-based telemedicine systems should be based on Internal Medicine cases and tailored to the specific local context (4).

Despite its evident advantages, the widespread adoption of telemedicine has been hindered by technical limitations at the point of care, regulatory policies, and limited reimbursement structures (1). However, the emergence of the COVID-19 pandemic has accelerated change in many areas and led to the rapid adoption of diverse telemedicine services. During this period, telemedicine has shown its potential to improve access to healthcare for patients with or without SARS-CoV-2 infection, while also ensuring the safety of patients and healthcare workers by maintaining physical distance (3, 4). Nevertheless, there is substantial evidence that shows the non-use and discontinued use of telemedicine. User-related factors, such as attitudes and technical literacy, are identified as key barriers to adoption along with technical aspects such as poor usability (4). Therefore, the systematic implementation of telemedicine should not only be based on technical feasibility, but also validated by evidence of real-world results and, ideally, robust evaluation.

In light of these considerations, the European Federation of Internal Medicine (EFIM) soundly recommends Internal Medicine societies and specialist physicians throughout Europe to take a proactive role in leading and influencing the development and application of telemedicine and digital technologies in healthcare. The purpose of this position paper is to outline the roles that telemedicine applications play in the practice of Internal Medicine in Europe.

## 3 Methods

The development of this position paper involved the participation and contribution of all the authors through a comparison process conducted remotely from July 2021 to December 2023. The primary methodology employed for this endeavor was the SWOT exercise with a Delphi panel. The Delphi method is a forecasting framework that involves multiple rounds of questionnaires sent to a panel of experts. Its application is deemed as efficient and simple, and often results in a consensus among a group of experts. In this particular instance, the authors of the paper qualified as experts in the field as they had applied telemedicine techniques directly and indirectly implemented within their respective national contexts (5–7).

The experts of Telemedicine Working Group collaboratively respond to the grand question “What role should Telemedicine play in the care of Internal Medicine patients?”

For each identified topic, a panel of experts was selected to encompass expertise in clinical, public health, health economics, and statistics domains. The panel assigned scores reflecting the perceived value, considering the balance between strengths and weaknesses, and the potential risks, considering the balance between threats and opportunities. These scores were evaluated on a Likert scale from −10, indicating minimum added value or risk, to +10, representing maximum added value or risk. All panel discussions were carried out remotely (8).

## 4 Results

### 4.1 Telemedicine SWOT analysis

A comprehensive checklist was developed by closely aligning the findings from the review of relevant literature on telemedicine practice with the outcomes derived from the Delphi analysis. Following the structure of the Delphi questionnaire, the checklist included factors that facilitate and hinder the implementation of telemedicine. The SWOT analysis is presented in Table 1 (5, 6, 9).

**Table 1:** Telemedicine SWOT analysis.

Strengths	Weaknesses
In remote patient/ home-monitoring of COVID-19 patients has facilitated outpatient care, early discharge, and recovery at home. no physical contact with patients minimizes the risk of exposure (especially during pandemic). willingness of healthcare systems to develop and adopt the technology platforms. different professionals in a variety of disciplines can conduct teleconsultations. higher versatility of schedules. hardware available in most centers. useful at different care levels. increased accessibility of primary, secondary, and tertiary healthcare. teleconsultation, telediagnosis, teleradiology, telepathology, etc., allows for remote assessment where access to expertise and infrastructure may not be available barriers to performing a complete physical examination.	lack of knowledge among professionals and confidence in the value of telemedicine. lack of patient health literacy, digital literacy. lack of physician digital literacy and skills. lack of interoperability of ICT systems. need for digital infrastructure. teleconsultations may not be fully integrated into the care process. lack of a humanization plan that includes telemedicine. need for more time to prepare the video consultation. increased risk in clinical decision making. need for a report at the end of the teleconsultation. absence of a developed legal framework. decentralization in the health system. inaccessible to patients do not have access to technology or who are illiterate. ease of access has resulted in more consultations for inquiry purposes rather than for consultation.
Opportunities	Threats
reduction in consultation time. reduction of geographical barriers and transportation costs. increased service portfolio and healthcare coverage. increased quality of care. optimization of clinical pathways. adequate referrals can be strengthened through telemedicine. environmental sustainability. longitudinal healthcare. reduction of visits to emergency department. reduction of risks in a pandemic situation.	aging population with low digital literacy and low eHealth literacy. low patient engagement. patient-physician relationship impairment. privacy of the patient, while communicating with a physician. fear of change (all stakeholders). fear of legal problems. fear of health professionals’ replacement with the use of ICT.

Substantial evidence now supports the strengths and opportunities associated with telemedicine. Telemedicine has been shown to reduce consultation time (10), eliminate unnecessary travel for both patients and healthcare professionals (11), facilitate healthcare delivery in remote areas (12), and contribute to cost savings (13). Integrating telemedicine into a well-coordinated care process has been demonstrated to improve health outcomes (14). In fact, patient-provider collaboration (“co-care”) and patient self-management (“self-care”) are not only an expression of patient-centeredness, they will also increase the cost-effectiveness of healthcare due to improved clinical outcomes and increased patient responsibilities and inputs.

Conversely, there is evidence highlighting the weaknesses and risks associated with telemedicine. Although the process of digitalization impacts approximately 90% of the healthcare sector, digital health extends beyond technological implementation and involves profound substantial cultural and social implications. It fundamentally alters the role of physicians and patients and the dynamics of their relationship. Patients now play an active role in the treatment process, fostering a patient-centric model where technology serves as a key tool for encouraging patient engagement and responsibility (15).

It is crucial to meticulously examine ethical issues in the delivery of telemedicine to ensure the confidentiality and security of patient information, address inefficiencies among physicians, and improve the overall quality of healthcare services. Ethical concerns related to telemedicine can be viewed from various perspectives, including technology, physician-patient relationships, data confidentiality and security, informed consent, and satisfaction of patients and their families with telemedicine services. Prioritizing ethical considerations in telemedicine is an essential aspect of ensuring the delivery of high-quality healthcare services (16, 17).

The physical examination performed by healthcare professionals, including medical doctors, has been a fundamental aspect of medical practice for centuries. This examination, involving sensory engagement, has been instrumental in enabling healthcare practitioners to assess the health status of their patients (18). Notably, research indicates that patients place high value on the physical examination not only for its perceived higher accuracy but also for its emotional attributes (19). Moreover, the diagnostic process is not solely based on a single episode of rational decision-making, instead, it involves continuous monitoring of the patient’s condition and subsequent adjustment of care (20). Telemedicine should be recognized as an alternative form of healthcare delivery that is distinct from traditional medical care. In this context, the interaction between technology and the local context holds significant importance (21).

In addition to the information presented in Table 1, there remains unexplored territory regarding the ethical dimensions of telemedicine. These include aspects related to the physician-patient relationship, data confidentiality and security, informed consent, and the satisfaction of both patients and caregivers.

Following the completion of the process, four scenarios were generated, considering various potential future developments.

## 5 Strengths and opportunities

### 5.1 Accelerated digitalization in Internal Medicine

During the SARS-CoV-2 coronavirus pandemic, telemedicine emerged as a natural and necessary solution to address global emergency healthcare needs (22–24). Telemedicine consultations, or teleconsultations (25–28), are valuable in diverse clinical scenarios, allowing for accurate differential diagnoses and appropriate treatment recommendations (29–31). Importantly, after such consultations, patients not only received medical advice but also benefited from e-prescriptions, e-referrals for further examinations (such as laboratory tests), and e-sick notes. This procedure significantly reduces the risk of infection for patients who would otherwise have to physically visit a healthcare center and wait in traditional waiting rooms for their appointments with doctors.

Examples of use-cases of evidence-based telemedicine applications in Internal Medicine include:

- Teletriage and remote consultations between patients and physicians in rural and remote areas or where mobility is an issue (32) or care for the older adult in their home environment, especially when living independently at home (33).
- In time-sensitive emergency care scenarios, where access to a specialist cannot be provided on-site within a safe timeframe, such as in the context of stroke care (34).
- Telemonitoring of chronic conditions, such as chronic heart failure and arrhythmias (35).
- Video consultations as part of long-term patient care (36).
- Remote consultations as a protection strategy during the COVID-19 pandemic (37).
- Additional self-directed care mechanisms described in a later section.

### 5.2 Digital literacy: a core skill for patients & clinicians

The process of digitalization requires digital health literacy, which is an extension of health literacy and uses an equivalent operational definition in the context of technology. Digital health literacy, or electronic health (e-health) literacy, focuses on an individual’s ability to access, understand, and engage with digital healthcare materials and technologies to contribute to quality of life (38). Technology solutions have the potential to promote health literacy. However, to be effective, health technology solutions must focus on functional and critical skills rather than building literacy and numeracy skills. Effective examples of functional and critical skills include operating the healthcare system, communicating with healthcare professionals, and sharing decision-making (37).

Stakeholders involved in telemedicine should also have adequate digital literacy and e-health literacy. Specifically, healthcare professionals need to develop specific competencies to effectively apply telemedicine to their routine practices. As digital health resources become more prevalent, the individual ability to interact with technology is to be assessed to ensure that the technology is appropriate for the intended audience (38–40).

Training of health care professionals should include (1):

- discussion of the individual stages of teleconsultations.
- patient interviews via telemedicine.
- examples of correct recommendations.
- attention to the alarm symptoms.
- sick notes issued after teleconsultations.
- Differences in internet access can also affect the quality and content of medical education (41, 42).

At a society level, educational campaigns should promote and support increased access to digital literacy and infrastructure necessary for successful eHealth solutions (43).

Interprofessional medical care or network medicine across healthcare settings can benefit from the development of eHealth competencies in physicians (44), advanced practice nurses, specialty nurses, physician assistants, and additional affiliated health professionals.

However, healthcare professionals also need to evaluate additional factors in telemedicine application, such as deployment costs at the point of care and high-speed Internet access for patients. Digital health inequity is defined as a systemic inequality that results from infrastructure disparities between countries and regions (45–49).

### 5.3 Telemedicine classification and modalities

To establish common definitions for the different typologies of telemedicine, Internal Medicine specialist physicians may distinguish them according to the methods of interaction employed, as following shown:

- According to its purpose: teleconsultation, telediagnosis, telemonitoring, telecare, teletraining, telerehabilitation.
- According to the technology employed: mobile health app, telephone, mail, videoconference, chat, messaging within the Electronic Health Record (EHR).
- According to the interlocutor: physician-patient, physician – physician, tele-training.
- According to the timing of execution: synchronous (interlocutors interact simultaneously), asynchronous (interlocutors interact at different times).

The methodologies in patient-physician interactions in telemedicine can be categorized into two main modalities: synchronous live and asynchronous interactions (32). However, academic studies comparing outcomes of asynchronous and synchronous care are still limited (50, 51).

#### 5.3.1 Synchronous live interactions

Synchronous live interactions involve real-time, instant exchanges between participants within a telemedicine environment. This mode of interaction is widely accepted and facilitates simultaneous transmission of information in both directions. This mode also allows healthcare professionals to evaluate patients face-to-face and gain crucial information about their care and disease status.

Examples of synchronous live interactions:

- Teleconsultations: between healthcare professionals or between a healthcare professional and a patient using synchronous information and communication technology platforms such as video, chat, and phone. Teleconsultations can be employed as an alternative to face-to-face consultations.
- Teletherapy: remote therapy sessions, such as physiotherapy, occupational therapy, psychology, and speech therapy, accomplished between a therapist and a patient through synchronous ICT communication.
- Remote monitoring: digital solutions, such as smartphone apps or web portals, to enable healthcare professionals to remotely monitor patient health data, such as blood pressure, electrocardiogram (ECG), and glucose levels. This technology makes it possible to intervene at the right timing and contributes to the prevention of hospitalization or urgent hospital admission. Remote monitoring has great potential in the continuous monitoring and prevention of exacerbation in chronic diseases. Remote monitoring is primarily asynchronous, but it can sometimes be combined with synchronous teleconsultations.

#### 5.3.2 Asynchronous interactions

Asynchronous interactions, or “store-and-forward” technology, facilitate the interaction of participants at separate time intervals in telemedicine. Asynchronous telemedicine services include various forms of communication, such as emails, secure text messaging, or services that allow both parties to engage at different times. This approach benefits healthcare professionals as they have the flexibility to review patient materials or communications on their own schedule. Asynchronous interactions enable patients to access healthcare services at their convenience in their preferred settings.

Asynchronous approaches are particularly relevant in fields such as dermatology, radiology, orthopedics, ophthalmology, and cosmetic surgery where image and video sharing are often required. However, there are also advantages in Internal Medicine consultations where an asynchronous approach can be utilized following a holistic patient-centered approach.

Examples of asynchronous approaches include:

- Remote patient monitoring (telemonitoring): includes registration, transmission, processing of body parameters such as vital signs and medical management through electronic systems. Wireless devices, wearable or implantable sensors, and medical apps can be integrated. Chronic diseases can be managed according to the patient’s needs. Most aspects are asynchronous, but synchronous elements, such as video consultations, can be integrated. Current innovations include the integration of Artificial Intelligence and Machine Learning algorithms for monitoring and early detection, e.g., in cardiac arrhythmias and hearth insufficiency (52).
- Remote interpretation telemedicine includes authorized access to healthcare data by healthcare professionals to interpret at any time and location.

#### 5.3.3 E-messaging

E-messaging, or chat-based interactions, involves exchanging messages via electronic devices such as tablets and mobile phones with the use of mobile networks and the Internet. Technologies employed for e-messaging include Short Message Services (SMSs) and applications such as FaceTime, Line, Messenger, WeChat, WhatsApp, and Viber. Approved and General Data Protection Regulation (GDPR)-compliant services (53) should be constantly used to secure transmission of patient personal health data, vital signs, physiologic data, diagnostic images, and self-reports to healthcare professionals. These technologies allow healthcare professionals to review and deliver consultations, diagnoses, and treatment plans at a later time, as well as support patient compliance, monitoring, prevention, treatment, and appointment reminders.

Privacy and data security are essential in e-messaging technologies. National Health Services (NHS) provided comprehensive guidelines for e-messaging services in Europe. Compliance with Europe’s General Data Protection Regulation ensures patient information and maintains data privacy (53, 54).

#### 5.3.4 Self-directed care mechanisms

Self-directed care mechanisms, which can be synchronous or asynchronous, include self-management that allows individuals to obtain healthcare information and schedule patient appointments at any time and location. In addition, self-management includes diagnostic tools, video tutorials, educational resources, and the ability to self-assess health indicators (55). Personal alarm systems, such as an alarm button or a wristband, enable patients to promptly contact response call centers in the event of a fall, personal injury, accident, or other critical emergencies (56). The following list provides examples of various telemedicine applications in the field of Internal Medicine (57–63).

- Complex chronic patient care during episodes of exacerbation.
- Hospitalization at home.
- Telemonitoring of vital signs in exacerbation.
- Video consultations with different specialists.
- Addressing uncertainties in treatment modalities for individuals with chronic conditions, such as health education and health literacy.
- New patients referred through teleconsultation, e.g., consultations with the primary care doctor related to analytical alterations, the treatment of chronic diseases.
- New patients evaluated with no physical examination.
- Periodic medical checks of stable chronic pathologies.
- Older adult patients with access restrictions.
- Intensive follow-up following hospital discharge.
- Individual or group training consultations via video call.

During teleconsultations, a standardized protocol is essential for conducting teleconsultations as it facilitates the acquisition of all relevant information required (64, 65). The next list summarizes teleconsultation steps:

#### 5.3.5 Pre-consultation

- Inform the patients about the necessary technical requirements for the consultation.
- Recommend the patients to take notes and have questions ready during the consultation.
- Specify estimated time and type of the consultation.
- Prepare the consultation by reviewing the clinical history and complementary tests.

#### 5.3.6 During the consultation

- Identify the patients. This is accomplished through either familiarity with the patients or by presenting the patients’ electronic health card or Identity Card card to the camera.
- Request consent for the consultation.
- Communicate messages in an orderly manner.
- Allow patients to express their doubts.
- Verify that the information has been fully comprehended.
- Review the agreements and alerts on possible warning signs and mode of action.
- Do not record too long video consultation hours.
- Prefer software with end-to-end encryption on videos.

#### 5.3.7 Post consultation

- Document that the consultation was accomplished by video.
- Document the relevant aspects of the consultation including the recommendations for further treatment, re-consultation, and/or referral to another health care provider.

In addition to standardized conduct of teleconsultation, specific warning signs should be carefully evaluated to protect patient safety and prevent the potential for reduced accuracy of remote visits compared to in-person visits. A summary of warning signs is shown in the following list (66, 67).

- Issues in understanding relevant medical information.
- Sudden worsening of clinical symptoms.
- Appearance of new symptoms that require physical examination.
- Signs of clinical instability or unexpected evolution.
- Need for hospital admission or emergency care.
- Need to communicate a poor prognosis or negative news.
- Situations that generate anxiety for the patient or the family.
- New patients with complex diagnoses.
- Uncooperative patients.

### 5.4 Enhancing the benefits of telemedicine applications

In the European region, harmonized guidance on the usage of telemedicine among specialist physicians is lacking. A telemedicine sharing protocol for European specialists does not exist at the time of this writing. Numerous countries, concentrated in particular in Southern Europe, had insufficient operational and legislative tools to rapidly introduce telemedicine services in outpatient specialist care (68).

Telemedicine can significantly reduce readmissions when monitoring patients with chronic diseases (60). However, the inability to conduct a complete physical examination during a teleconsultation is potentially a major barrier to the development of remote consultation services (69, 70). The application of telemedicine devices, such as e-stethoscopes or video cameras, and artificial intelligence algorithms will increase the possibilities of telemedicine in the future. Such development leverages existing experience from fields such as teledermatology, which has successfully integrated digitally enabled clinical examination of the skin (71). Additionally, expressions of empathy can support trust during a patient-physician encounter, and the frontier of digital empathy may be paramount in sustaining such constructs in telemedicine visits (72).

A hybrid model could be considered for long-term care in both primary care and specialist care and would also need evaluation over the long term. This model allows alternating in-person appointments at the health facilities and teleconsultation appointments (73, 74). A similar model employing telephone follow-up visits has been used in many clinical trial protocols, significantly reducing the number of health center visits and hospitalizations (71).

#### 5.4.1 Noncommunicable chronic disease and multimorbidity care

Various academic studies have demonstrated that telemedicine is not inferior to in-person consultations in the management of patients with heart failure, hypertension, and diabetes (75) Telemedicine can effectively prevent exacerbations, hospitalizations, and disease progression (76). However, the efficacy of telemedicine compared to in-person visits depends on the specific medical field and the patient characteristics. In addition, real-time interactive consultation may be more beneficial than delayed consultation (74).

Monitoring therapy adherence via telemedicine tools is essential (77) Telemedicine tools include a range of devices, such as continuous vital sign monitors, digital reminders, ingestible sensors, video observation, and smartphone applications. Trials evaluating the effectiveness of telemedicine tools have been conducted in China, India, Italy, Belarus, and the United States (78).

#### 5.4.2 Aging in place with telemedicine

The identification of older adult patients with mild cognitive impairment or dementia, who may be at a high risk of acute conditions, can be eased by mobile technologies and telemedicine. Telemedicine solutions should be customized for the older adult to be user-friendly and potentially automated (79).

The introduction of telemedicine can reduce the financial burden on public expenditures related to the older adult segment (80). Telemedicine improves the reach and efficiency of public healthcare resources and encourages collaboration among healthcare professionals and patients/caregivers. In addition, this approach contributes to reduced hospitalization rates and associated risks such as falls, healthcare-associated infections, compensation claims, and improved treatment adherence (67).

Appropriate utilization of emergency services and optimal ward utilization can also benefit of such technological enhancement because various preventive and real-time monitoring actions can be performed remotely, eliminating the necessity for patients to physically visit a healthcare facility (81, 82).

This is extremely useful especially for older adult and frailty population, who are the most responsible for inappropriate healthcare services utilization (83, 84).

This could also apply to territorial integration between acute hospital wards and intermediate care facilities, such as rehabilitation or palliative care structures. Their timely coordination is paramount in easing the burden of discharge process in hospital wards (85–90).

Nevertheless, the efficacy of telemedicine depends on individual digital literacy levels and the development of reliable digital infrastructures (70).

Older adult people would especially benefit from telemedicine, as the continuous monitoring of vital parameters can slow the progression or exacerbation of chronic conditions (67). Telemedicine can also build a sense of community, especially for isolated patients. In conclusion, the integration of human intelligence and telemedicine can produce increasingly personalized medicine, identification of risk factors and extrapolation of patient risk curves. Telemedicine has also proved to be effective in contrasting geriatric depression (91).

### 5.5 Roles and responsibilities of other healthcare members

Health Information Technologies (HIT) have the potential to improve the quality of interprofessional and team care coordination, benefiting patients as well as healthcare. Specifically, HIT can support shared decision-making, access to care information (such as open notes) and care services (such as synchronous remote telehealth services), and health education. HIT enhances team care similar to that of another member of the healthcare team, automating routine or tedious tasks so that human agents can focus on providing humanized healthcare. Beyond routine tasks such as scheduling or administrative aspects of care, HIT can further evolve to enable previously unfeasible models of care, such as hospital-at-home care or intensive remote monitoring in selected conditions. Augmented intelligence provides humans with actionable data and information, enhancing human intelligence and decision-making (15).

When planning for novel care models, it is essential to engage HIT developers and clinical informaticians with healthcare training, such as physicians, pharmacists, nurses, and other relevant professionals. In addition, the involvement in the design process of patients and their advocates can also be beneficial. This inclusive approach guarantees the ethical and equitable design of healthcare systems (92, 93).

### 5.6 Methods to enhance clinical decision-making in telemedicine

Despite growing political support for telemedicine systems, their standardization within clinical practice has been hampered by concerns about their effectiveness, cost-effectiveness, and user acceptance (70, 94).

Telemedicine makes it possible to provide healthcare services regardless of geographical constraints. Telemedicine and its associated technologies enable us to switch from the movement of individuals to the flow of information (16).

Telemedicine possesses several positive attributes, such as reduced entry barriers, established health services, integration of primary and specialty care, delivery of care through smart devices in patient homes, patient preference, and convenience. These factors are particularly significant for fragile and vulnerable populations (95). In addition, telemedicine favors the integration of local health systems and hospitals by facilitating communication between internal specialists and general practitioners.

### 5.7 Challenges and benefits of health technology assessment application to telemedicine technologies

Telemedicine offers benefits in various cases by easing the load on healthcare infrastructure and personnel and ensuring timely and adequate care to patients who face mobility issues and are geographically distant from appropriate medical facilities (96). However, additional telemedicine dimensions requiring evaluation concern the ethical and social aspects of telemedicine such as the patient-physician relationship, data confidentiality and security, informed consent, and patient and caregiver satisfaction. Most suitable telemedicine devices should be carefully selected, procured, and connected with medical professionals for evaluation. While technology has the potential to improve patient access and health outcomes, not all technological innovations can achieve their intended purposes (97, 98). Thus, the investigation of different telemedicine technologies is necessary to prioritize the ones that are efficient and impactful. The Health Technology Assessment (HTA) process (99–103) plays a crucial role in evaluating the adequacy of telemedicine technologies. The HTA carries out a systematic assessment to determine the suitability and effectiveness of various telemedicine approaches (99).

### 5.8 Ethical and legal considerations

Various types of regulations are touched upon in the jurisdiction of European Law, including primary and secondary regulations, as well as soft law in the form of guidelines and communications issued by the European Commission. With reference to primary law, the Treaty on the Functioning of the European Union (TFEU) plays a central role (104). Article 56 of the TFEU prohibits any restrictions on the freedom to provide services, while Article 57 of the TFEU defines the very notion of service. Medical care falls within the scope of the Treaty as it regulates the free movement of services. As for secondary law, Regulation (EU) 2016/679 and Directive 95/46/EC, known as General Data Protection Regulation (GDPR), are the main reference regulations. Regulation (EU) 2016/679 concerns the protection of personal data and their free movement, while Directive 95/46/EC pertains to health data and genetic data and emphasizes the rights of patients in cross-border healthcare (105, 106). Furthermore, this Directive aims to provide clear regulation for the phenomenon known as “medical tourism.”

Recitals 19 and 20 of the preambles already impose an obligation to inform patients receiving cross-border healthcare about the applicable rules. Upon request, healthcare professionals are also required to provide specific information about the healthcare benefits they offer and the treatment options available. Directive 2011/24/EU further clarifies the information obligations of healthcare professionals under Article 4 (107). According to this directive, healthcare professionals should offer relevant information to support individual patients in making informed decisions, including details on treatment options, availability, quality, and safety of healthcare services, as well as prices for specific benefits. At the same time, Article 4 of Directive 2011/24/EU requires Member States to ensure that healthcare professionals on their territory apply the same fee structure for patients from other Member States as for domestic patients in comparable medical situations. If no comparable prices exist for domestic patients, healthcare professionals should charge a price based on objective and non-discriminatory criteria. This approach is explained by the need to establish standards for telemedicine services to preserve patient’s and medical personnel’s safety and protection.

In summary, this approach is consistent with solutions planned at the EU level. In 2018, the European Commission announced ongoing efforts to provide citizens with secure access to high-quality digital health and welfare services. A communication on the digital transformation of health and social care has been published, outlining three key areas for further action. The first area focuses on actions to ensure secure access and sharing of health data for citizens. The European Commission plans to establish an e-health digital service infrastructure that allows for the exchange of e-prescriptions and patient data between healthcare professionals in order to facilitate access to cross-border healthcare. Development is underway to establish a European electronic health record exchange format accessible to all EU citizens. The second area stresses the importance of better data for research, disease prevention, and personalized healthcare. The third area highlights the use of digital tools to empower citizens and provide person-centered care. Digital services should be scaled up to enable individuals to manage their health effectively. Consequently, the proposed telemedicine standards align perfectly with these adopted assumptions (108).

## 6 Weaknesses and threats

### 6.1 Limitations of telemedicine

Specific limitations may prevent the adoption, implementation, and expansion of telemedicine and its supporting technologies. Extensive training is required to familiarize patients with video teleconsultations and the use of assistive technologies. Physicians also require targeted technical, clinical, and communication training tailored to their specific subspecialty needs. Limited access to broadband and internet facilities is a significant barrier, especially in remote areas and under-resourced settings (11). Reliable broadband access is essential for telemedicine services, but its quality is often inadequate in rural clinics and for patients residing in such areas.

Legal restrictions and ambiguity in permissible practices in telemedicine have created a cautious attitude among telemedicine professionals. In addition, certain medical conditions are not adequately addressed within existing healthcare legislation. The pricing structure for virtual consultations and video surveillance in hospitals remains unclear, leaving questions as to whether they will be fully reimbursed or classified as shorter visits at a discounted rate. Physician licensing and telemedicine infrastructures pose additional concerns, especially in resource-scarce settings.

Telemedicine cannot replace many essential medical procedures and is not universally accessible to all patients. Various patient groups may be further marginalized by healthcare technologies, for example, people whose language(s) are not concordant with those of the telemedicine clinician, people with disabilities (109, 110), may be excluded or face challenges in using telemedicine. The effectiveness of telemedicine depends on its successful integration into the existing hospital and healthcare system within a local context, adequate preparation and training of medical professionals, and patient awareness and acceptance of telemedicine tools.

### 6.2 A vision for the future of telemedicine in Internal Medicine

To speculate on the future of telemedicine, various future scenarios emerge from the EFIM Telemedicine Working Group’s overview of academic literature (111). The most probable scenario implies the emergence of a hybrid system where telemedicine augments traditional healthcare services, enhancing efficiency and adaptability to evolving patient care needs in a local context. The goals and measured outcomes of such hybrid models would be to ensure high-quality, accessible, equitable, efficient care, which holds the entire pathway of care services to a similar standard of health outcomes, regardless of the level of technology integration into healthcare services.

Four possible scenarios are expected to emerge within the hybrid system by considering the evolving behavior of different stakeholders.

#### 6.2.1 Scenario 1: best-case scenario

In the best-case scenario, all aspects related to telemedicine have significantly improved since 2022. The use of telemedicine has increased significantly, with physicians significantly incorporating it into their practices. Research and development efforts have reduced barriers to use and increased technology efficiency and security. User-friendly platforms have been developed, making patients and physicians increasingly rely on telemedicine. In addition, innovative approaches have explored the expansion of telemedicine across different medical specialties by managing virtual and face-to-face components of appointments. Overall, telemedicine is widely adopted, well-understood, and proven to be efficient and effective in this scenario.

#### 6.2.2 Scenario 2: worst-case scenario

In the worst-case scenario, all aspects surrounding telemedicine have deteriorated since 2022. Certain variables have reverted to pre-COVID-19 practices, and significant investments in research and development have not materialized. Consequently, little progress has been made in making telemedicine technology more accessible, secure, or inclusive to minority groups. As a result, patients and physicians have become discouraged, and telemedicine is seen as a last resort rather than an integral part of healthcare.

#### 6.2.3 Scenario 3: physician pushback scenario

This scenario is similar to the best-case scenario, except physicians are more reluctant to adopt telemedicine. This scenario may arise because of changes in physician perceptions over time or because telemedicine placed additional burdens on physicians. However, ongoing research and development efforts may reverse among physicians and make them more proactive about telemedicine. Lower barriers to use and high patient willingness to engage have the potential to move this scenario toward a best-case situation.

#### 6.2.4 Scenario 4: effort to improve scenario

Scenario 4 is similar to Scenario 2 worst-case scenario but differs in terms of important ongoing research and development efforts. However, barriers to access remain high and patient willingness to engage with telemedicine is low. Consequently, this scenario is likely to head toward a worst-case situation (80).

According to these hypotheses, the primary factors influencing future scenarios in healthcare will be the propensity of physicians and patients to adopt new technologies to redefine the doctor-patient relationship. Regardless of whether future scenarios are positive or negative, the existence and inevitability of technological advancements will remain. However, it is important to note that the development of technology alone is not sufficient to facilitate the establishment of a new patient care model.

### 6.3 EFIM position on telemedicine and recommendations

Built on the scenario analysis, the EFIM Working Group proposes the following recommendations for telemedicine implementation:

- Clinical Care Standards: Guarantee that clinical care standards for telemedicine are consistent traditional office visit standards, comprising all aspects of diagnosis and treatment decisions.
- Clinical Judgment: Use clinical judgment in establishing the scope and extent of telemedicine applications, especially in the diagnosis and treatment of specific patients and chronic conditions.
- Authorization and Reimbursement: Approve and refund live interactive telemedicine in Internal Medicine in a way similar or equivalent to traditional in-person visits, subject to commitment to the principles outlined.
- Definition of Roles and Responsibilities: Define the roles, anticipations, and responsibilities of providers involved in Internal Medicine Telemedicine, including source and remote locations.
- Development of Models of Care: Move forward models of care in telemedicine, where Internal Medicine specialists, patients, primary care providers, and other healthcare team members work together to improve the value of healthcare delivery in a collaborative way.
- Compliance with Technical Standards: Maintain appropriate technical standards in the telemedicine delivery process, at the source and remote location.
- Investigation of Improvement Methods: Consider ways to extend telemedicine utility, including the use of patient explainers, community resources, providers, ancillary tests, and additional technologies.
- Quality Assurance Processes: Apply quality assurance processes for telemedicine care delivery models, with the intent of catching process measurements, patient outcomes, and patient/provider experiences.
- Data Management Time Recognition: Acknowledge the period required for data management, quality processes, and other aspects of care delivery related to telemedicine within a value-based care delivery model.
- Compliance with Professional and Ethical Standards: Warrant accurate compliance with professional and ethical standards in the use of telemedicine services and equipment ensuring patient access, quality, and value of care.
- Billing Transparency: Promote billing transparency for telemedicine services, and support patients, providers, and others to understand payer reimbursements throughout the entire process.
- Research and Impact Assessment: Recognize the probable rapid expansion of telemedicine use in Internal Medicine and broader telehealth applications, highlighting the necessity of further research to evaluate the impact and outcomes of these technologies.

## 7 Conclusion

Further investigation is necessary to evaluate the optimal use of telemedicine in the field of Internal Medicine. Based on existing scientific evidence, the European Federation of Internal Medicine (EFIM) recommends increased utilization of these innovative methods to provide adequate care for complex patients with multiple chronic conditions. Given the ongoing epidemiological shift and rapid technological advancements, EFIM believes that the significant adoption of telemedicine is critical in providing comprehensive care for Internal Medicine patients.

## Author contributions

FP: Conceptualization, Writing – review & editing, Writing – original draft. MF: Investigation, Writing – original draft. SK: Software, Writing – review & editing. KK: Data curation, Writing – review & editing. TL: Methodology, Writing – original draft. ISG: Supervision, Writing – review & editing. SS: Formal analysis, Writing – original draft. MR: Formal analysis, Writing – original draft. AS: Project administration, Writing – original draft. FR: Writing – original draft, Writing – review & editing. CD: Project administration, Writing – original draft. AV: Resources, Writing – original draft. VB: Validation, Writing – review & editing. NM: Resources, Writing – review & editing. DD: Funding acquisition, Writing – review & editing. RGH: Visualization, Writing – review & editing.
